# The Effects of the oxLDL/β2GPI/anti‐β2GPI Complex on Macrophage Autophagy and its Mechanism

**DOI:** 10.1002/iid3.70058

**Published:** 2024-11-07

**Authors:** Qianqian Wu, Guiting Zhang, Ting Wang, Hong Zhou

**Affiliations:** ^1^ Department of Transfusion Medicine, Nanjing Drum Tower Hospital Medical School of Nanjing University Nanjing China; ^2^ Department of Clinical Laboratory, Nanjing Drum Tower Hospital Medical School of Nanjing University Nanjing China; ^3^ Department of Clinical Laboratory and Hematology, School of Medicine Jiangsu University Zhenjiang China

**Keywords:** autophagy, macrophages, oxLDL/β2GPI/anti‐β2GPI complex, PI3K/AKT/mTOR, TLR4/NF‐κB

## Abstract

**Background:**

Previous research has established that the oxidized low‐density lipoprotein/β2‐glycoprotein I/anti‐β2‐glycoprotein I antibody (oxLDL/β2GPI/anti‐β2GPI) complex can stimulate macrophages to secrete molecules associated with atherosclerosis (AS), such as monocyte chemotactic protein 1 (MCP‐1), tissue factor (TF), and tumor necrosis factor‐α (TNF‐α). This complex also enhances the uptake of oxLDL, thereby accelerating foam cell formation through the Toll‐like receptor‐4/nuclear factor kappa B (TLR4/NF‐κB) pathway. Given the critical role of macrophage autophagy in the instability of vulnerable atherosclerotic plaques, it is imperative to investigate whether the oxLDL/β2GPI/anti‐β2GPI complex influences macrophage autophagy in AS. This study aims to elucidate the effects and underlying mechanisms of the oxLDL/β2GPI/anti‐β2GPI complex on macrophage autophagy in AS.

**Methods:**

Experiments were conducted using murine macrophage RAW264.7 cells and the human monocytic cell line THP‐1. Western blot analysis was employed to determine the expressions of autophagy‐associated markers and signaling pathway proteins. Autophagosomes were detected through mRFP‐GFP‐LC3 adenoviral transfection and transmission electron microscopy (TEM).

**Results:**

Treatment of macrophages with the oxLDL/β2GPI/anti‐β2GPI complex resulted in decreased expressions of Beclin1 and LC3 proteins, alongside an upregulation of SQSTM1/P62 protein expression. Additionally, there was a reduction in the number of autophagosomes and autolysosomes. An increase in the phosphorylation levels of phosphoinositide‐3‐kinase (PI3K), protein kinase B (AKT), and mammalian target of rapamycin (mTOR) was also observed. Notably, the expressions of autophagy‐associated markers were partially restored when the TLR4/NF‐κB and PI3K/AKT/mTOR pathways were inhibited by their respective inhibitors.

**Conclusions:**

Our findings indicate that the oxLDL/β2GPI/anti‐β2GPI complex inhibits macrophage autophagy in AS via the TLR4/NF‐κB and PI3K/AKT/mTOR signaling pathways.

**Abbreviations**
anti‐β2GPI: anti‐β2‐glycoprotein I
aPL: antiphospholipid antibody
APS: antiphospholipid syndrome
AS: atherosclerosis
NF‐κB: nuclear factor kappa B
oxLDL: oxidized low density lipoproteins
PAMPs: pathogen‐associated molecular patterns
PI3K/AKT/mTOR: phosphoinosmde‐3‐kinase/protein kinase B/mammalian target of rapamycin
SLE: systemic lupus erythematosus
TLR4: toll‐like receptor 4
β2GPI: β2‐glycoprotein I

## 1 Introduction

Antiphospholipid syndrome (APS) is an acquired autoimmune disease characterized by the presence of high titers of antiphospholipid antibodies (aPL), which can easily cause arteriovenous thrombosis. Anti‐β2GPI is the main component in aPL, and its recognition target β2GPI is the main antigen in APS [1, 2]. It's been reported that patients with APS have an accelerated development of atherosclerosis (AS) [3]. AS is a cardiovascular disease characterized by inflammation, lipid accumulation, local neovascularization and apoptosis in the arterial wall, leading to the death and disability worldwide [4].

Studies have reported that autophagy exerts anti‐atherosclerotic effects in the early stages of atherosclerosis by reducing inflammation and inhibiting disease progression [5]. However, as plaque advances, autophagy may also play a detrimental role. Specifically, autophagy is suppressed in endothelial cells, smooth muscle cells, and macrophages, leading to insufficient clearance of misfolded proteins and damaged organelles, as well as increased inflammation [6]. Conversely, excessive autophagy can degrade a significant portion of the cytoplasm and organelles, culminating in autophagic cell death, plaque instability, and acute cardiovascular events. The effects of autophagy on atherosclerosis are multifaceted and may be protective or harmful depending on factors such as the concentration of oxidants, duration of exposure, stage of plaque development, and type of cells involved [7]. Mouse models of atherosclerosis have demonstrated that deficiencies in macrophage autophagy can accelerate the progression of the disease [5, 8].

Autophagy serves as a protective mechanism for cells under conditions such as nutrient deficiency, hypoxia, oxidative stress, protein aggregation, and endoplasmic reticulum (ER) stress. These conditions trigger autophagy by activating autophagy‐related signaling molecules and initiating the formation of the protein Atg1 (ULK1) [9]. Subsequently, a double‐layered membrane structure forms to encapsulate aggregated proteins, damaged organelles, and intracellular pathogens. This membrane elongates and closes to form a bilayer vesicle known as the autophagosome [10], which ultimately fuses with the lysosome to form autolysosomes. The encapsulated contents are then degraded and recycled [11, 12]. During autophagy, Beclin1 is a key signaling molecule responsible for recruiting other autophagy proteins to the pre‐autophagy structure [13]. LC3 plays a crucial role in autophagy; its soluble form (LC3‐I) undergoes processing and lipidation, becoming part of the autophagosome membrane as LC3‐II [14]. LC3‐II is widely recognized as an important marker for the initiation and extension of autophagy [15]. SQSTM1/P62, associated with the accumulation of damaged proteins and organelles, is significantly upregulated in atherosclerotic plaques, linking impaired autophagy to cytotoxicity, inflammasome activation, and apoptosis [16]. Additionally, chloroquine (CQ) is a late‐stage inhibitor of autophagy, primarily by inhibiting the fusion of autophagosomes with lysosomes, thereby preventing autophagosome degradation [17].

The phosphoinositide‐3‐kinase/protein kinase B/mammalian target of rapamycin (PI3K/AKT/mTOR) pathway is an autophagy inhibitory pathway closely related to cell survival, proliferation, and differentiation [18]. Activation of PI3K leads to the phosphorylation of AKT at two key residues, T308 and S473 [19, 20]. AKT directly regulates the mammalian target of rapamycin complex 1 (mTORC1), a key sensor of nutrient signaling that regulates cell metabolism, translation, and cytokine response [21, 22]. Recently, Wang et al. discovered that in cases of subarachnoid hemorrhage, FGF‐2 can suppress neuronal autophagy through the modulation of the PI3K/Akt signaling pathway [23].

Toll‐like receptor 4 (TLR4), a member of the toll‐like receptor (TLR) family, is extensively expressed on the surface of macrophages and various other cell types. Upon activation by pathogen‐associated molecular patterns (PAMPs), TLR4 triggers a signal transduction cascade, playing a pivotal role in immune defense. Notably, TLR4 is highly expressed in human atherosclerotic plaques and its expression can be upregulated by LDL [24, 25]. Compared to individuals with stable coronary atherosclerosis, those with vulnerable coronary atherosclerotic plaques exhibit elevated TLR4 expression in circulating monocytes [26]. The interplay between TLR and autophagy is complex, involving various downstream signaling molecules associated with activated innate immune receptors [27]. Increasing evidence indicates that macrophages act as intermediaries between autophagy and immunity [28].

Our previous research has shown that the oxLDL/β2GPI/anti‐β2GPI complex promotes the formation of peritoneal macrophages and increases the expression of pro‐inflammatory factors in BALB/c mice through the TLR4/NF‐κB pathway, thereby promoting the pathological process of atherosclerosis (AS) [29]. However, it remains unclear whether the oxLDL/β2GPI/anti‐β2GPI complex affects macrophage autophagy in AS and whether the TLR4/NF‐κB and PI3K/AKT/mTOR signaling pathways are involved in the suppression of autophagy. Therefore, for the first time, we aimed to investigate the effects of the oxLDL/β2GPI/anti‐β2GPI complex on macrophage autophagy in AS and its underlying mechanisms. Furthermore, in light of the high‐titer anti‐β2GPI antibodies present in patients with antiphospholipid syndrome (APS), along with oxLDL/β2GPI and β2GPI/anti‐β2GPI complexes, we designated these components as a control group for oxLDL/β2GPI/anti‐β2GPI in this study.

## 2 Materials and Methods

### 2.1 Cell Culture

The human monocytic cell line THP‐1 and murine macrophage RAW264.7 cells were purchased from the Shanghai Institutes for Biological Sciences (Shanghai, China). THP‐1 cells were cultured in RPMI‐1640 (Sigma, St. Louis, MO, USA) supplemented with 10% FBS (BI, Israel) at 37°C in a humidified atmosphere containing 5% CO_2_. THP‐1 cells were exposed to 100 ng/mL phorbol 12‐myristate 13‐acetate (PMA, ENZO Biochem) for 48 h for monocyte differentiation. In all experiments, THP‐1 cells were differentiated into THP‐1 cell‐derived macrophages before further analysis. RAW264.7 cells were cultured in DMEM (Gibco BRL, Grand Island, NY, USA) supplemented with 10% FBS (Wisent, Montreal, Canada) at 37°C in a humidified atmosphere containing 5% CO_2_.

### 2.2 Experimental Treatment

Macrophages were stimulated with various combinations of oxLDL (50 μg/mL), β2GPI (100 μg/mL) and anti‐β2GPI (100 μg/mL), including culture medium, oxLDL, oxLDL/anti‐β2GPI complex, oxLDL/β2GPI complex, β2GPI/anti‐β2GPI complex, and oxLDL/β2GPI/anti‐β2GPI complex. In certain experiments, macrophages were pretreated with 5 µM TLR4 inhibitor TAK242 (InvivoGen) [29], 20 µM NF‐κB inhibitor PDTC (Sigma) [29], 10 µM autophagy inhibitor chloroquine (CQ, Sigma) [17], or 50 µM PI3K pathway inhibitor LY294002 (Cell Signaling Technology) according to the product instruction.

### 2.3 Western Blot Analysis

The expressions of AKT, p‐AKT, LC3, P62, mTOR, BECLIN1, p‐mTOR, PI3K and p‐PI3K at the protein level were determined using Western blot analysis. Briefly, cells exposed to different stimuli were lysed in RIPA buffer (P0013K, Beyotime Institute of Biotechnology), collected and centrifuged at 12,000 rpm for 10 min at 4°C. Equal amounts of proteins were subjected to SDS‐PAGE and then transferred onto PVDF membranes (Bio‐Rad Laboratories, Inc., Hercules, CA, USA). Subsequently, the membranes were blocked in 5% fat‐free milk at room temperature for 2 h, followed by incubation with primary antibodies against β‐actin (1:4000, Bioward, Nanjing, Jiangsu, China), p‐AKT (1:2000, Cell Signaling Technology), LC3, BECLIN1, SQSTM1/P62, AKT, mTOR, p‐mTOR, PI3K and p‐PI3K(1:1000, Cell Signaling Technology, Beverly, MA, USA) overnight at 4°C. The membranes were washed with TBST for three times and then incubated with horseradish peroxidase (HRP)‐conjugated goat anti‐rabbit secondary antibodies (1:4000, Bioworld) at room temperature for 1 h. Immunoreactive bands were visualized using electrochemiluminesence detection (GE Healthcare, Chicago), and the band intensity was analyzed using the Gel‐Pro analyzer.

### 2.4 mRFP‐GFP‐LC3 Adenoviral Transfection

The cells were seeded into a 24‐well plate, and according to the manufacturer's instructions, the mRFP‐GFP‐LC3 adenovirus (HanBio Technology, Shanghai, China) transfection was performed when a cell confluence of 60% was achieved. The multiplicity of infection (MOI) values of RAW264.7 cells and THP‐1 cell‐derived macrophages were 300 and 500, respectively. After 6 h of transfection, the culture medium was replaced by the adenovirus‐free medium. On the next day, the cells were exposed to different stimuli for 24 h. After that, the cells were washed twice using PBS and then fixed. The images were obtained to assess the intracellular autophagy flow using the confocal laser scanning microscope (LSM 880 with Airyscan; Zeiss, Dublin, CA, USA) by adjusting the 10× eyepiece and 60× objective lens. Bright fluorescent spots could be observed under the lens, in which the yellow dots (RFP^+^ and GFP^+^) represented autophagosomes and the red dots (RFP^+^ and GFP^‐^) represented autolysosomes. The autophagosome/autolysosome fluorescence microscopy image quantification was conducted manually.

### 2.5 Transmission Electron Microscopy (TEM)

TEM was used to detect the autophagosomes. After above‐mentioned treatments, the cells were collected and fixed in pre‐cooled electron microscopy fixative (2.5% glutaraldehyde) overnight. Subsequently, the cells were stained with 1.5% osmium tetroxide and 4% uranium. Then the cells were embedded in epoxy resin. Finally, 0.1 μm thin sections were stained and viewed in TEM (HITACHI‐HT7700, Japan). The autophagosome quantification in TEM was conducted manually.

### 2.6 Statistical Analysis

Data were presented as mean ± SEM. Difference between two groups was assessed using Student's unpaired *t*‐test (two tailed), and multiple group comparisons were conducted using ANOVA. All experiments were repeated at least three times independently. Statistical significance was defined as *p* < 0.05.

## 3 Results

### 3.1 oxLDL/β2GPI/anti‐β2GPI Complex Inhibits the Expressions of Autophagy‐Associated Proteins in Macrophages

**Figure 1:**
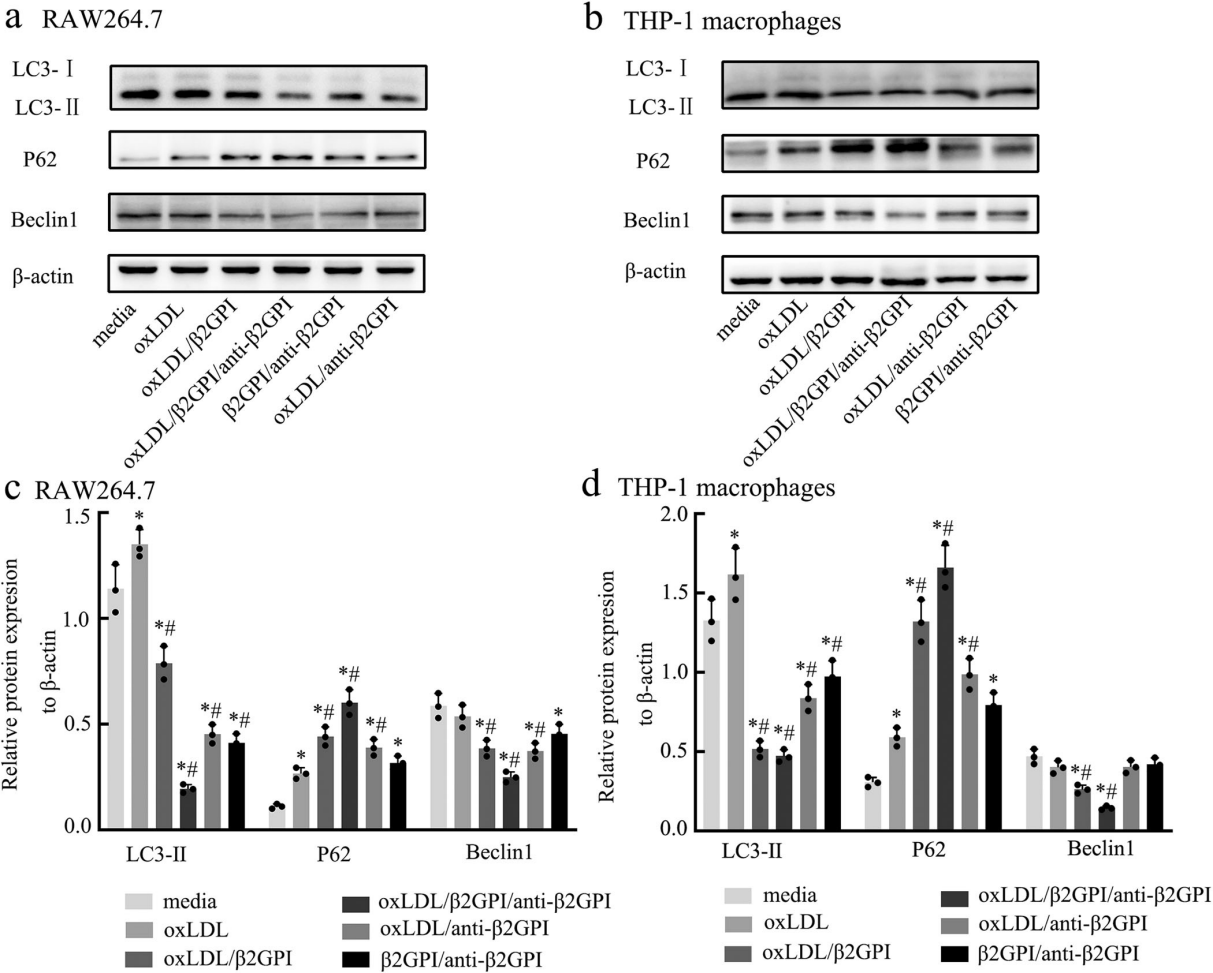
Expressions of autophagy‐associated proteins in macrophages after exposure to different stimuli. RAW264.7 and THP‐1 cell‐derived macrophages were treated with culture medium, oxLDL (50 μg/mL), oxLDL/anti‐β2GPI (50, 100 μg/mL) complex, oxLDL/β2GPI complex (50, 100 μg/mL), β2GPI/anti‐β2GPI complex (100, 100 μg/mL), or oxLDL/β2GPI/anti‐β2GPI complex (50, 100, 100 μg/mL) for 24 h. (a, b) Western blot analysis of the expressions of autophagy‐associated proteins. (c, d) Semi‐quantitative analysis of protein levels of LC3, P62 and Beclin1 in macrophages. The detection of WB was carried out using six‐well plates, with 1.2 × 10^6^ cells per well. Data from three independent experiments are expressed as mean ± SEM (*n* = 3). All groups are compared with the media group, *p < 0.05 versus the media group. All groups are compared with the oxLDL group, ^#^ *p* < 0.05 versus the oxLDL group.

Murine macrophage RAW264.7 cells and THP‐1 cell‐derived macrophages were treated with different stimuli for 24 h, after which the cells were collected to detect autophagy‐associated proteins. LC3‐II is widely recognized as an important marker for the initiation and extension of autophagy [15], while Beclin1 is a key signaling molecule for autophagy initiation [13]. As shown in Figure 1a,b, the expression levels of LC3‐II protein in the oxLDL/β2GPI/anti‐β2GPI group were significantly lower than those in the media group and oxLDL group for both types of macrophages (*p* < 0.05). Similarly, a decrease in LC3‐II protein levels was observed in the oxLDL/β2GPI group, oxLDL/anti‐β2GPI group, and β2GPI/anti‐β2GPI group, although these changes were not as pronounced as in the oxLDL/β2GPI/anti‐β2GPI group. For Beclin1 protein expression, a decrease was noted in the oxLDL/β2GPI group and oxLDL/β2GPI/anti‐β2GPI group in both types of macrophages (*p* < 0.05 vs. media, *p* < 0.05 vs. oxLDL, Figure 1a–d). In the oxLDL/anti‐β2GPI group and β2GPI/anti‐β2GPI group, a decrease in Beclin1 expression was observed in RAW264.7 cells, but no significant change was observed in THP‐1 cell‐derived macrophages, which may be related to species‐specific differences.

SQSTM1/P62 is known to be an autophagic substrate, and its accumulation indicates a deficiency in autophagic flux [16]. As depicted in Figure 1a–d, the expression of SQSTM1/P62 was significantly increased in the oxLDL/β2GPI/anti‐β2GPI group (*p* < 0.05 vs. media). Similar changes were observed in other groups, although the upregulation was not as remarkable as in the oxLDL/β2GPI/anti‐β2GPI group. Compared to the oxLDL group, the oxLDL/β2GPI/anti‐β2GPI group also exhibited a statistically significant increase in the expression of P62 protein in both types of macrophages (*p* < 0.05, Figure 1a–d). Overall, these findings suggest that the oxLDL/β2GPI/anti‐β2GPI treatment decreases macrophage autophagy.

### 3.2 oxLDL/β2GPI/anti‐β2GPI Complex Reduces the Autophagosomes and Blocks Autophagic Flux in Macrophages

The detection of autophagosomes and autophagic flux in macrophages following exposure to various stimuli was conducted using several methods. LC3, a protein expressed on the autophagosome membrane, plays a crucial role in this process. During autophagy, the mRFP‐GFP‐LC3 fusion protein, initially dispersed in the cytoplasm, translocates to the autophagosome membrane, forming bright fluorescent spots observable via laser confocal microscopy. These spots are indicative of autophagosomes (yellow dots) and autolysosomes (red dots).

**Figure 2:**
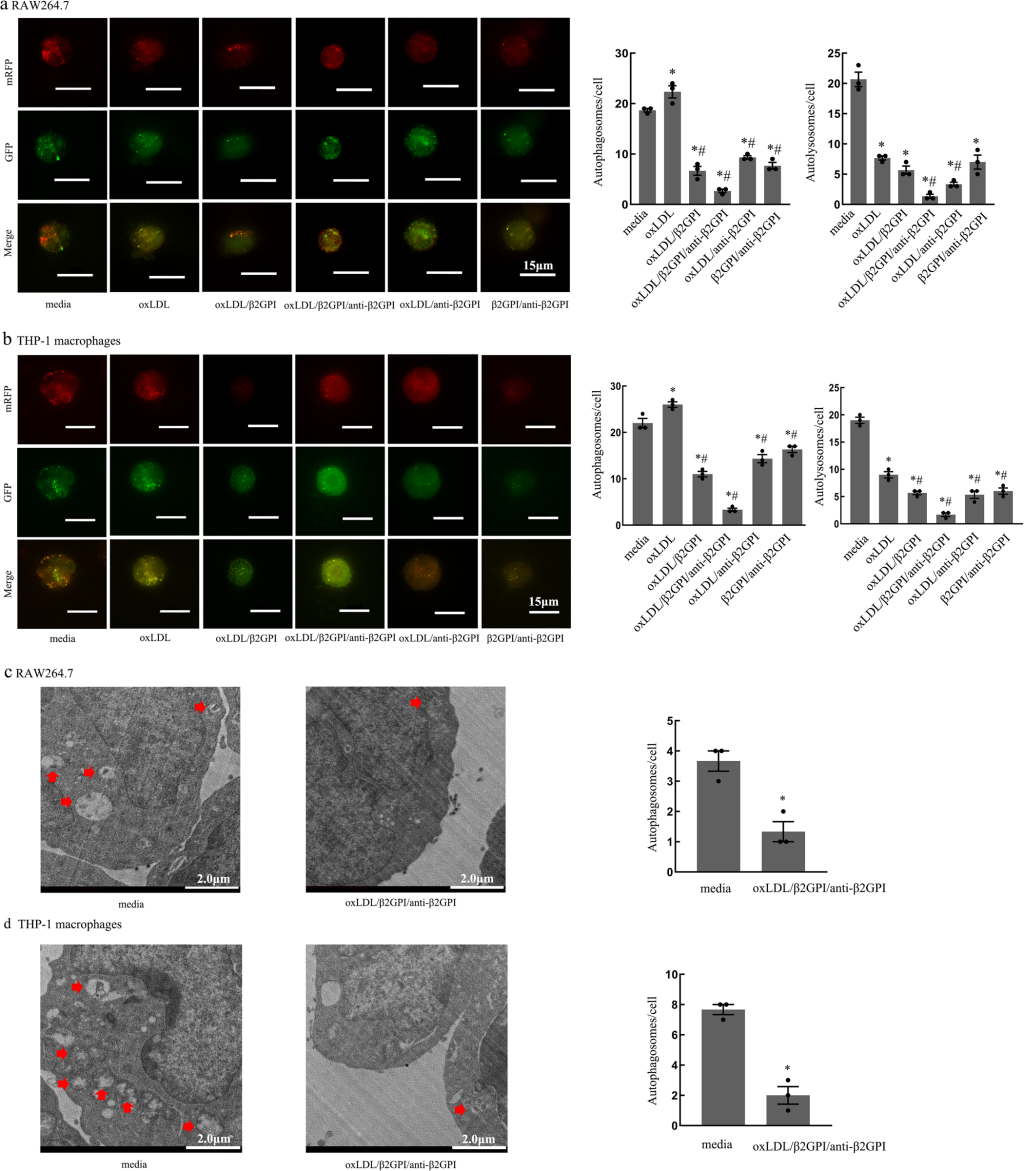
Autophagosomes and autolysosomes in macrophages after exposure to different stimuli. RAW264.7 and THP‐1 cell‐derived macrophages were transfected with mRFP‐GFP‐LC3 adenovirus and treated with culture medium, oxLDL (50 μg/mL), oxLDL/anti‐β2GPI complex (50, 100 μg/mL), oxLDL/β2GPI complex (50, 100 μg/mL), β2GPI/anti‐β2GPI complex (100, 100 μg/mL), or oxLDL/β2GPI/anti‐β2GPI complex (50, 100, 100 μg/mL) for 24 h. (a, b) Representative images (magnification, ×600) and quantification of autophagosomes and autolysosomes in different groups0 (scale bars: 15 µm). Each experiment was conducted independently three times, and three representative images were selected for capture during each repetition. (c, d) TEM detection and quantification of autophagosomes (magnification, ×3000) in macrophages (red arrow, scale bars: 2 μm). The detection of autophagy dual‐label adenovirus was conducted using 24‐well plates, with 1.25 × 10^5^ cells per well. The detection of TEM was implemented using six‐well plates, with 1.2 × 10^6^ cells per well. Data from three independent experiments are expressed as mean ± SEM (*n* = 3). All groups are compared with the media group, *p < 0.05 versus the media group. All groups are compared with the oxLDL group, ^#^ *p* < 0.05 versus the oxLDL group.

In RAW264.7 cells, as shown in Figure 2a, the oxLDL/β2GPI/anti‐β2GPI group exhibited a significant reduction in the number of autophagosomes and autolysosomes compared to the media group (*p* < 0.05). Similar decreases were observed in the oxLDL/β2GPI, oxLDL/anti‐β2GPI, and β2GPI/anti‐β2GPI groups (*p* < 0.05), although these reductions were less pronounced than in the oxLDL/β2GPI/anti‐β2GPI group. When compared to the oxLDL group, all other groups showed a reduction in the number of autophagosomes (*p* < 0.05), with the most significant changes in the oxLDL/β2GPI/anti‐β2GPI group. However, only the oxLDL/β2GPI/anti‐β2GPI and oxLDL/anti‐β2GPI groups showed a decrease in the number of autolysosomes compared to the oxLDL group (*p* < 0.05).

In THP‐1 macrophages, as depicted in Figure 2b, the oxLDL/β2GPI/anti‐β2GPI group also showed a significant decrease in the number of autophagosomes and autolysosomes compared to both the media and oxLDL groups (*p* < 0.05). The oxLDL/β2GPI, oxLDL/anti‐β2GPI, and β2GPI/anti‐β2GPI groups exhibited reductions in autophagosomes and autolysosomes (*p* < 0.05), though these reductions were less significant than those in the oxLDL/β2GPI/anti‐β2GPI group.

TEM is currently the gold standard for detecting autophagy, as it allows for the observation of autophagosomes characterized by a bilayer membrane vacuole structure containing cytoplasmic components such as mitochondria and endoplasmic reticulum. Figure 2c,d illustrates autophagosomes in macrophages detected by TEM. The number of autophagosomes in macrophages treated with oxLDL/β2GPI/anti‐β2GPI was significantly reduced compared to the culture medium group (*p* < 0.05, Figure 2c,d), suggesting an inhibition of autophagy.

### 3.3 The Effect of CQ on Macrophage Autophagy Mediated by oxLDL/β2GPI/anti‐β2GPI Complex

**Figure 3:**
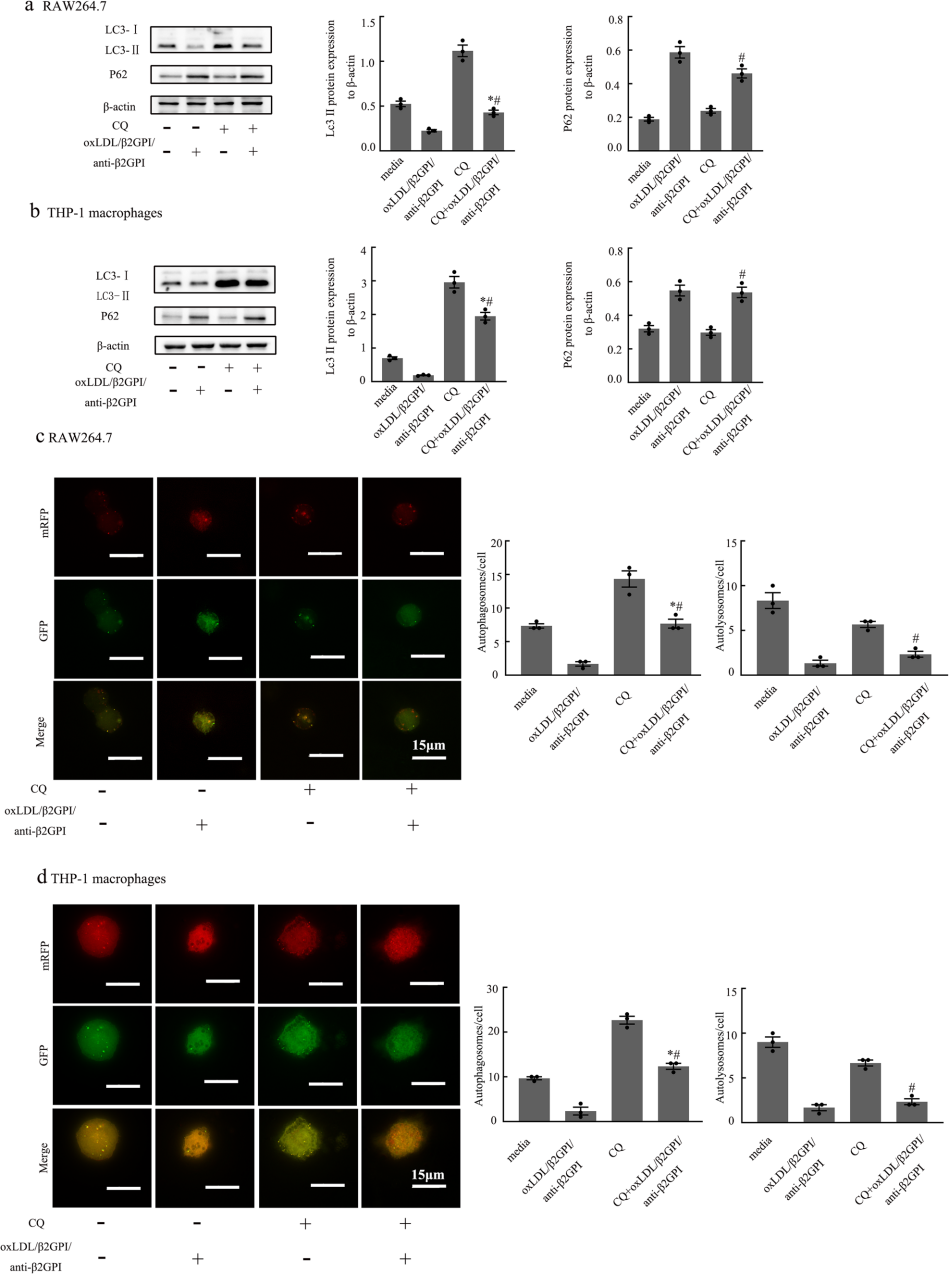
Effects of CQ on autophagy‐associated proteins and autophagic flux in macrophages. RAW264.7 and THP‐1 cell‐derived macrophages were pretreated with CQ (10 μM) for 3 h, and then exposed to different stimuli for 24 h. (a, b) Western blot analysis of the autophagy‐associated proteins and the semi‐quantitative analysis of protein levels of LC3 and P62 in macrophages. (c, d) Representative images (magnification, ×600) and quantification of autophagosomes and autolysosomes in different groups (scale bars: 15 µm). Each experiment was conducted independently three times, and three representative images were selected for capture during each repetition. The detection of WB was carried out using six‐well plates, with 1.2 × 10^6^ cells per well. The detection of autophagy dual‐label adenovirus was conducted using 24‐well plates, with 1.25 × 10^5^ cells per well. Data from three independent experiments are expressed as mean ± SEM (*n* = 3). oxLDL/β2GPI/anti‐β2GPI with CQ pretreatment is compared with the oxLDL/β2GPI/anti‐β2GPI group, *p < 0.05 versus the oxLDL/β2GPI/anti‐β2GPI group. oxLDL/β2GPI/anti‐β2GPI with CQ pretreatment is compared with the CQ group, ^#^ *p* < 0.05 versus the CQ group.

Autophagy is a dynamic process, and to accurately determine the stage at which the oxLDL/β2GPI/anti‐β2GPI complex inhibits macrophage autophagy, we pretreated the cells with the lysosomal inhibitor chloroquine (CQ) at a concentration of 10 µM. Subsequently, we observed changes in autophagic markers. As shown in Figure 3, the LC3‐II protein expression and the number of autophagosomes were significantly decreased in the oxLDL/β2GPI/anti‐β2GPI + CQ treatment group compared to the CQ‐treated group (*p* < 0.05, Figure 3a–d). This suggests that the oxLDL/β2GPI/anti‐β2GPI complex inhibits the formation of autophagosomes at the early stage of autophagy. Additionally, the expression of P62 protein was significantly increased, and the number of autolysosomes was markedly decreased in the oxLDL/β2GPI/anti‐β2GPI + CQ treatment group compared to the CQ group (*p* < 0.05, Figure 3a–d). This indicates that the oxLDL/β2GPI/anti‐β2GPI complex blocks autophagic flux at the late stage. Furthermore, pretreatment with CQ led to an increase in LC3‐II protein expression and the number of autophagosomes in the oxLDL/β2GPI/anti‐β2GPI + CQ group compared to the oxLDL/β2GPI/anti‐β2GPI complex group alone (*p* < 0.05, Figure 3a–d). We speculate that this is because CQ inhibits the fusion of autophagosomes with lysosomes, preventing their degradation and resulting in their accumulation. We speculate that it is because CQ can inhibit the combination of autophagosomes and lysosomes, resulting in the inability of autophagosomes to be degraded and thereby causing their accumulation and increase.

### 3.4 TLR4 and NF‐κB Molecules Participate in Macrophage Autophagy Inhibited by oxLDL/β2GPI/anti‐β2GPI Complex

**Figure 4:**
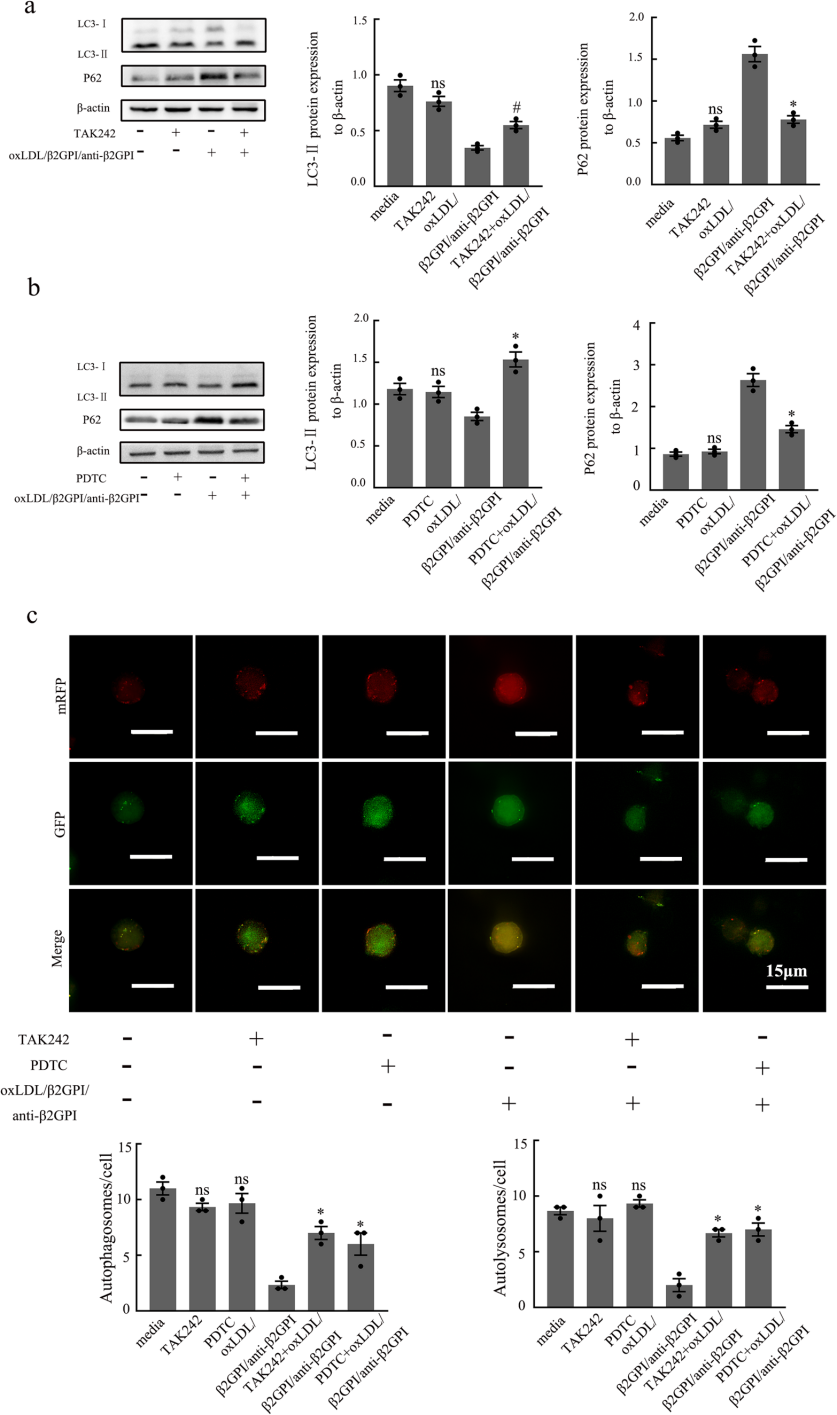
Effects of different stimuli on the expressions of autophagy‐associated proteins and autophagic flux in RAW264.7 cells. RAW264.7 cells were pretreated with TAK242 (5 μM) or PDTC (20 μM), and then exposed to different stimuli for 24 h. (a, b) Western blot analysis of the autophagy‐associated proteins and the semi‐quantitative analysis of protein levels of LC3, P62 and Beclin1 in macrophages. (c) Representative images (magnification, ×600) and quantification of autophagosomes and autolysosomes in different groups (scale bars: 15 µm). Each experiment was conducted independently three times, and three representative images were selected for capture during each repetition. The detection of WB was carried out using six‐well plates, with 1.2 × 10^6^ cells per well. The detection of autophagy dual‐label adenovirus was conducted using 24‐well plates, with 1.25 × 10^5^ cells per well. Data from three independent experiments are expressed as mean ± SEM (*n* = 3). oxLDL/β2GPI/anti‐β2GPI with TAK242 or PDTC pretreatment is compared with the oxLDL/β2GPI/anti‐β2GPI group, *p < 0.05 versus the oxLDL/β2GPI/anti‐β2GPI group. TAK242 or PDTC group is compared with the media group, ^#^ *p* < 0.05 versus the media group, and ns means no statistical significance.

Our previous research has confirmed that during the formation of foam cells, the TLR4/NF‐κB signaling pathway in macrophages is activated by the oxLDL/β2GPI/anti‐β2GPI complex [27]. In the present study, we aimed to examine the effects of TLR4 inhibitor TAK242 (5 µM) and NF‐κB inhibitor PDTC (20 µM) on macrophage autophagy. As shown in Figure 4, there were no significant differences in autophagy‐associated proteins and autophagosomes between the inhibitor (TAK242 or PDTC) group and the culture medium group (*p* > 0.05, Figure 4a–c), suggesting that these signaling pathway inhibitors alone cannot mediate macrophage autophagy. However, when macrophages pretreated with TAK242 were exposed to the oxLDL/β2GPI/anti‐β2GPI complex, there was a significant increase in LC3‐II protein expression, a decrease in P62 expression, and a restoration in the number of autophagosomes and autolysosomes (*p* < 0.05 vs. oxLDL/β2GPI/anti‐β2GPI, Figure 4a–c). This indicates that the inhibition of TLR4 can restore macrophage autophagy that is otherwise inhibited by the oxLDL/β2GPI/anti‐β2GPI complex. Similarly, the oxLDL/β2GPI/anti‐β2GPI + PDTC group exhibited comparable changes (*p* < 0.05 vs. oxLDL/β2GPI/anti‐β2GPI, Figure 4a–c), indicating that NF‐κB inhibition via PDTC can also restore macrophage autophagy inhibited by the oxLDL/β2GPI/anti‐β2GPI complex.

### 3.5 oxLDL/β2GPI/anti‐β2GPI Complex Enhances the Activity of PI3K/AKT/mTOR Pathway in Macrophages

**Figure 5:**
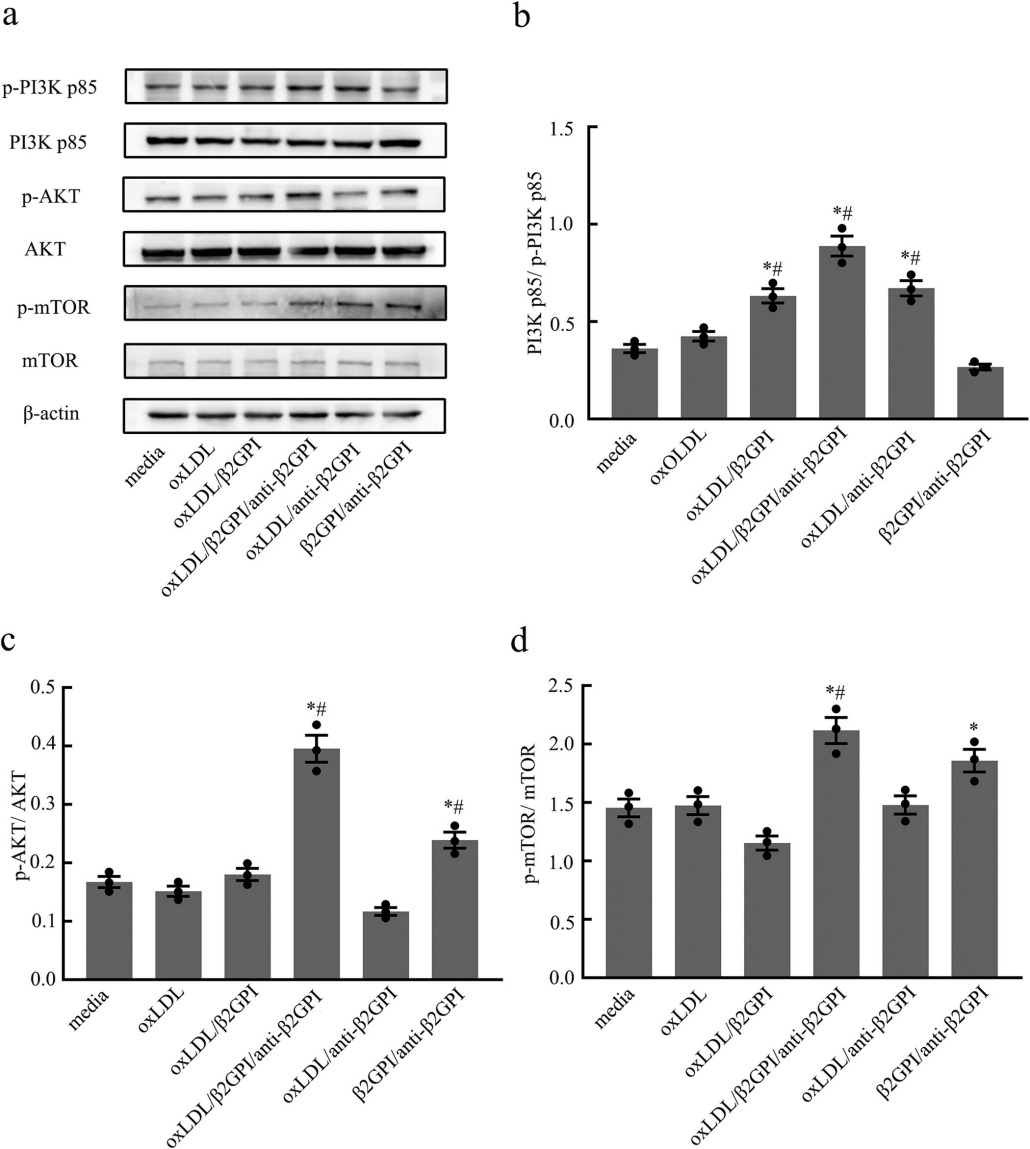
Effects of different stimuli on the expressions of p‐PI3K p85, p‐AKT and p‐mTOR at the protein level in RAW264.7 cells. RAW264.7 cells were treated with culture medium, oxLDL (50 μg/mL), oxLDL/anti‐β2GPI complex (50, 100 μg/mL), oxLDL/β2GPI complex (50, 100 μg/mL), β2GPI/anti‐β2GPI complex (100, 100 μg/mL), or oxLDL/β2GPI/anti‐β2GPI complex (50, 100, 100 μg/mL) for 24 h. (a) Western blot analysis of the phosphorylation of PI3K p85, AKT, and mTOR. (b–d) Semi‐quantitative analysis of protein levels of p‐PI3K p85, p‐AKT, and p‐mTOR. The detection of WB was carried out using six‐well plates, with 1.2 × 10^6^ cells per well. Data from three independent experiments are expressed as mean ± SEM (*n* = 3). All groups are compared with the media group, *p < 0.05 versus the media group, all groups are compared with the oxLDL group, ^#^ *p* < 0.05 versus the oxLDL group.

The PI3K/AKT/mTOR signaling pathway is known to inhibit autophagy and is intricately linked to cell survival, proliferation, and differentiation. To determine whether this pathway is implicated in the inhibition of macrophage autophagy by the oxLDL/β2GPI/anti‐β2GPI complex, we conducted Western blot analysis to measure the expression of phosphorylated PI3K, AKT, and mTOR proteins in macrophages subjected to various stimuli. Our findings revealed a significant increase in the phosphorylation levels of PI3K p85, AKT, and mTOR in the oxLDL/β2GPI/anti‐β2GPI complex group compared to both the culture medium group and the oxLDL group (*p* < 0.05, Figure 5a–d). In contrast, the phosphorylation changes of these signaling pathway proteins were not consistent across other groups. These results suggest that the oxLDL/β2GPI/anti‐β2GPI complex can activate the PI3K/AKT/mTOR signaling pathway.

### 3.6 LY294002 Can Restore the Expressions of Autophagy‐Associated Proteins Blocked by oxLDL/β2GPI/anti‐β2GPI Complex in Macrophages

**Figure 6:**
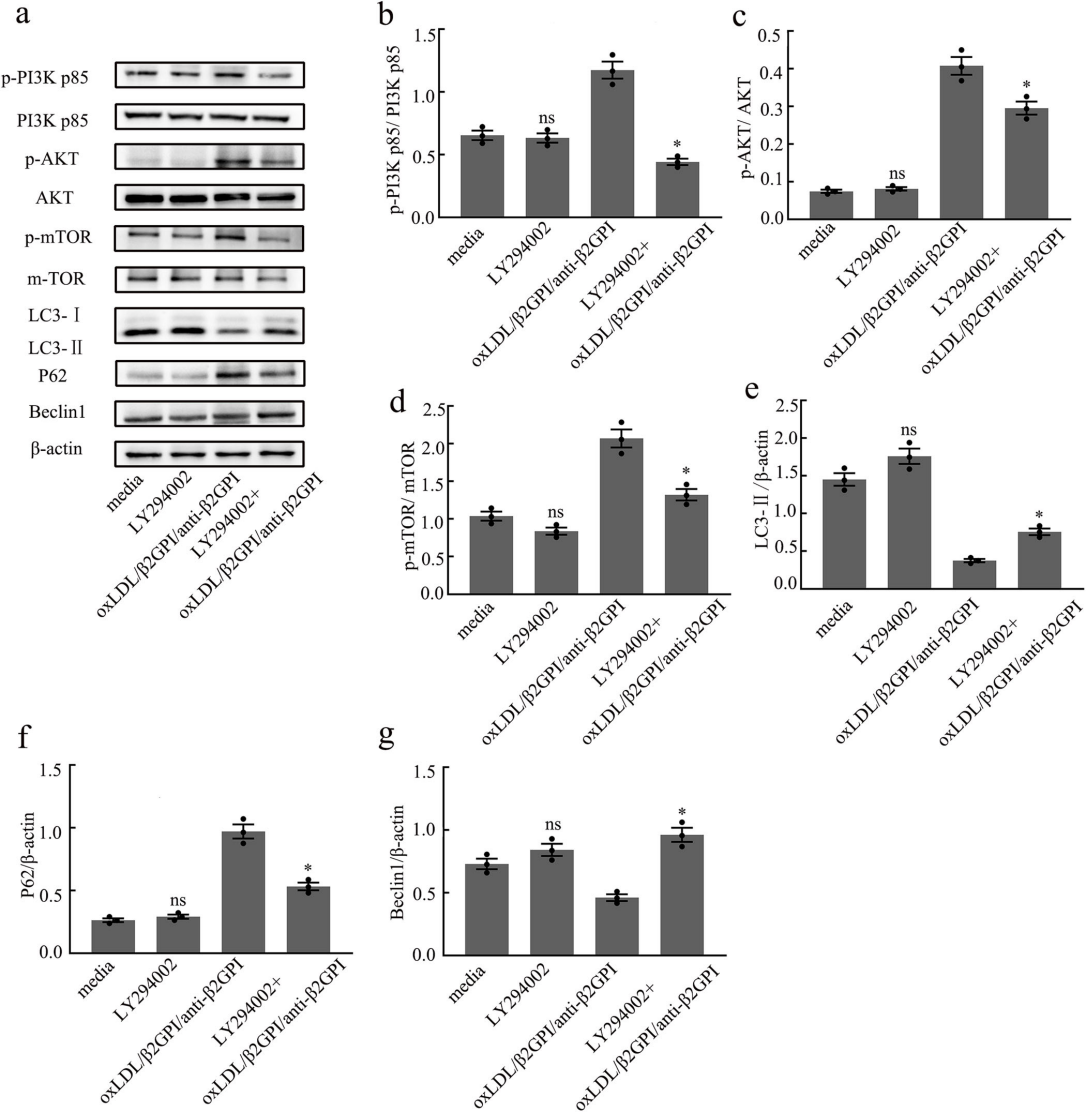
Effects of LY294002 on autophagy‐associated proteins in RAW264.7 cells inhibited by oxLDL/β2GPI/anti‐β2GPI complex. RAW264.7 cells were pretreated with LY294002 (50 μM) and then exposed to different stimuli for 24 h. (a) Western blot analysis of the autophagy‐associated proteins and PI3K pathway molecules. (b–g) Semi‐quantitative analysis of protein levels of LC3, P62, Beclin1, p‐PI3K p85, p‐AKT, and p‐mTOR in macrophages. The detection of WB was carried out using six‐well plates, with 1.2 × 10^6^ cells per well. Data from three independent experiments are expressed as mean ± SEM (*n* = 3). oxLDL/β2GPI/anti‐β2GPI with LY294002 pretreatment is compared with the oxLDL/β2GPI/anti‐β2GPI group, *p < 0.05 versus the oxLDL/β2GPI/anti‐β2GPI group. LY294002 group is compared with the media group, ^#^ *p* < 0.05 versus the media group, and ns means no statistical significance.

To further investigate the role of the PI3K/AKT/mTOR pathway in macrophage autophagy inhibited by the oxLDL/β2GPI/anti‐β2GPI complex, we pretreated macrophages with LY294002 (50 μM) for 1 h, followed by exposure to the corresponding stimuli for 24 h. Our results showed no significant difference in the expression of autophagy‐associated proteins and PI3K/AKT/mTOR pathway proteins between the LY294002 group and the culture medium group (*p* > 0.05, Figure 6a–g). This lack of difference may be attributed to the level of autophagic flux in the steady state of the cells, suggesting that the low flux might obscure the detection of changes in autophagic flux upon treatment with a PI3K inhibitor. However, in the oxLDL/β2GPI/anti‐β2GPI group pretreated with LY294002, there was a significant downregulation in the phosphorylation levels of PI3K, AKT, and mTOR, along with a notable reduction in the changes of LC3, P62, and Beclin1 protein expressions induced by oxLDL/β2GPI/anti‐β2GPI treatment (*p* < 0.05 vs. oxLDL/β2GPI/anti‐β2GPI, Figure 6a–g). These findings indicate that the PI3K/AKT/mTOR pathway is involved in the inhibition of macrophage autophagy by the oxLDL/β2GPI/anti‐β2GPI complex.

## 4 Discussion

APS patients frequently develop AS, a condition believed to be associated with elevated titers of aPL antibodies, particularly anti‐β2GPI antibodies [30]. β2GPI serves as the primary target antigen for these anti‐β2GPI antibodies. The interaction between β2GPI domain V and oxLDL, a significant risk factor for AS, leads to the formation of the oxLDL/β2GPI complex, which has been demonstrated to promote AS progression in APS patients [1, 31]. During the formation of this complex, domains I and/or IV of β2GPI become exposed and are subsequently recognized by anti‐β2GPI autoantibodies, resulting in the formation of the oxLDL/β2GPI/anti‐β2GPI complex [31].

Our research group has previously demonstrated that the oxLDL/β2GPI/anti‐β2GPI complex can enhance the expression of the pro‐inflammatory factor TNF‐α, the adhesion molecules VCAM‐1 and ICAM‐1, as well as the production of reactive oxygen species (ROS) and apoptosis in endothelial cells [32, 33]. Additionally, this complex has been shown to promote the proliferation and migration of smooth muscle cells and to impede reverse lipid transport [34]. Furthermore, it can induce macrophages to secrete a greater quantity of atherosclerosis‐associated molecules, including MCP‐1, TF, and TNF‐α, and increase the uptake of oxLDL, thereby accelerating the formation of foam cells [29, 35]. Despite these findings, the impact of the oxLDL/β2GPI/anti‐β2GPI complex on macrophage autophagy in AS and the underlying mechanisms remain largely unexplored. Therefore, for the first time, we aimed to investigate the effects of the oxLDL/β2GPI/anti‐β2GPI complex on macrophage autophagy and its underlying mechanisms in AS.

Autophagy is a highly conserved cellular process responsible for the removal or recovery of long‐lived proteins and damaged organelles, playing a crucial role in cell survival, differentiation, development, and homeostasis [36]. In the context of AS, mild oxidative stress can activate autophagy, thereby enhancing the clearance of damaged organelles and aiding in cellular recovery. Conversely, impaired autophagy leads to the accumulation of damaged mitochondria, increased production of ROS, and elevated levels of lipofuscin [37]. Experimental studies on Ldlr^‐/‐^ mice fed a Western diet have demonstrated that macrophage autophagy deficiency promotes plaque necrosis, macrophage apoptosis, and oxidative stress in advanced stages of AS [38]. Furthermore, recent research analyzing macrophages extracted from blood samples of patients with cardiovascular disease has revealed significant downregulation of autophagy‐related genes LC3 and Atg5, accompanied by a marked increase in TNF‐α levels [39].

This study found that the complexes oxLDL/β2GPI, oxLDL/β2GPI/anti‐β2GPI, oxLDL/anti‐β2GPI, and β2GPI/anti‐β2GPI could inhibit macrophage autophagy. This inhibition was evidenced by the downregulation of autophagy‐associated protein LC3‐II and RFP^+^GFP^+^ spots, as well as the upregulation of P62. Among these complexes, oxLDL/β2GPI/anti‐β2GPI caused the most significant changes. However, autophagy is a dynamic process, and it is crucial to observe not only autophagy formation but also autophagy degradation. To investigate the specific mechanism underlying the oxLDL/β2GPI/anti‐β2GPI‐inhibited macrophage autophagy, we pretreated cells with the autophagy inhibitor CQ. The results showed that the expression of LC3‐II protein in the oxLDL/β2GPI/anti‐β2GPI group treated with CQ was lower compared to the CQ alone group, indicating that oxLDL/β2GPI/anti‐β2GPI inhibits the formation of autophagosomes in the early stage of autophagy. However, the P62 protein levels in the oxLDL/β2GPI/anti‐β2GPI group treated with CQ exhibited no significant difference compared to the group treated with oxLDL/β2GPI/anti‐β2GPI alone, but were significantly elevated compared to the CQ alone group. This suggests that the oxLDL/β2GPI/anti‐β2GPI complex disrupts degradation during the late stage of autophagy, leading to a defect in autophagy flux and blocking the smooth progression of autophagy. This strengthens the inhibition of autophagy within macrophages, transforming it into defective autophagy. This defect might account for the high prevalence of atherosclerosis in patients with antiphospholipid syndrome in later stages. Autophagy is originally a stress‐protective mechanism of the organism. Under normal conditions, macrophages can form a double‐layer membrane structure to encapsulate excessive lipoproteins and degrade them with the assistance of lysosomes, thereby protecting cells from damage. However, the oxLDL/β2GPI/anti‐β2GPI complex inhibits autophagy within macrophages, causing them to lose their protective role. Simultaneously, it blocks autophagy flux and leads to autophagy dysfunction, potentially accelerating the development of atherosclerosis. Kazuhiro Ishimaru et al. have studied the aortic autophagy degradation process in Sphk2 mice and found that the exacerbation of atherosclerosis in Sphk2 mice may also be related to autophagy deficiency in plaque macrophages [40]. However, in our experimental outcomes, the increase in P62 protein expression in the group treated with CQ alone was not detected in either RAW264.7 cells or THP‐1 cells. However, a reduction in autophagic lysosomes was observed under the confocal microscope. We speculate that this discrepancy may be attributed to differences between the two detection methods. The Western blot approach might have failed to detect the accumulation of P62 in the CQ‐treated group due to the early time point selected for detection, which could have resulted in a low level of P62 protein accumulation. Alternatively, it is possible that the accumulated P62 protein at the detected time point had already undergone degradation.

TLR4 can be activated by LPS, leading to the production of pro‐inflammatory mediators that destroy pathogens. The pro‐inflammatory activity of TLR4 is also implicated in pathological responses to endogenous ligands under chronic inflammatory conditions, such as autoimmune diseases, ankylosing spondylitis (AS), and neurodegenerative diseases [41]. Our previous research has demonstrated that during the formation of foam cells, the oxLDL/β2GPI/anti‐β2GPI complex can increase the expression of TLR4 and NF‐κB in macrophages [29], induce apoptosis in endothelial cells [32], and affect the proliferation, migration, and apoptosis of smooth muscle cells via the TLR4 pathway [34, 42]. Additionally, TLR4 has been reported to regulate autophagy in various tissues, including the monocyte‐macrophage system [19]. This led us to speculate whether the TLR4/NF‐κB pathway is involved in the inhibition of macrophage autophagy by the oxLDL/β2GPI/anti‐β2GPI complex. Our previous studies confirmed that the oxLDL/β2GPI/anti‐β2GPI complex activates the TLR4/NF‐κB signaling pathway in macrophages [29]. In the current study, we pretreated cells with TAK242 (a TLR4 inhibitor) and PDTC (an NF‐κB inhibitor) and observed changes in autophagy‐associated markers. The addition of TAK242 or PDTC partially alleviated the inhibition of autophagy in macrophages. Based on these findings, we hypothesize that the oxLDL/β2GPI/anti‐β2GPI complex may inhibit macrophage autophagy through the TLR4/NF‐κB pathway. Similarly, it has been reported that Hirsutella sinensis mycelium can regulate autophagy in alveolar macrophages via the TLR4/NF‐κB signaling pathway [43].

Recent studies have demonstrated that the PI3K/Akt/mTOR signaling pathway is essential for orchestrating inflammatory responses via TLR4/NF‐κB [44, 45]. The PI3K signaling pathway, a downstream effector of various cell surface receptors, regulates cell proliferation, survival, and apoptosis [46]. Activation of TLR4 results in the recruitment of PI3K by facilitating the entry of junction molecules into the receptor complex. Our research found that oxLDL/β2GPI/anti‐β2GPI could enhance the phosphorylation levels of PI3K, AKT, and mTOR, thereby activating the PI3K/AKT/mTOR pathway. Given that oxLDL/β2GPI/anti‐β2GPI can transmit signals into cells through the transmembrane protein receptor TLR4, it can be speculated that PI3K, as a downstream component of the cell surface receptor, receives and responds to these signals. Upon activation, PI3K further stimulates the activation of mTOR via the AKT pathway. This, in turn, inhibits ULK1 of the autophagy pathway, leading to a blockade in autophagosome formation and resulting in negative feedback regulation. The PI3K/AKT/mTOR signaling pathway is highly complex, characterized by multiple levels of feedback inhibition; further experimental evidence is necessary to validate this hypothesis. Similarly, Huang CY et al. discovered that Hispolon has the ability to inhibit oxidative stress‐mediated ER stress‐induced apoptosis and autophagy by regulating the TLR4/PI3K/Akt/mTOR and Keap1/Nrf2/HO‐1 pathways [47].

To further investigate the role of the PI3K/AKT/mTOR pathway in oxLDL/β2GPI/anti‐β2GPI‐inhibited autophagy in macrophages, we pretreated the cells with the PI3K pathway inhibitor LY294002. This pretreatment resulted in a decrease in phosphorylated PI3K, AKT, and mTOR proteins, indicating that the PI3K/AKT/mTOR signaling pathway was effectively inhibited. Concurrently, we observed that LY294002 pretreatment mitigated the changes in LC3, P62, and Beclin1 protein expressions induced by the oxLDL/β2GPI/anti‐β2GPI complex. These findings suggest that LY294002 can alleviate the degree of autophagy inhibition in macrophages mediated by the oxLDL/β2GPI/anti‐β2GPI complex. Consequently, the inhibition of macrophage autophagy was significantly weakened by suppressing PI3K/AKT/mTOR signal transduction, underscoring the involvement of the PI3K/AKT/mTOR signaling pathway in oxLDL/β2GPI/anti‐β2GPI‐inhibited autophagy of macrophages. This observation aligns with the report by Mingxue Zhou et al. which suggests that inhibiting the activation of the PI3K/AKT/mTOR pathway could be an effective strategy to enhance autophagy [38]. Additionally, studies have demonstrated that inhibiting the PI3K/AKT/mTOR pathway in cells can selectively and effectively induce autophagy, thereby reducing macrophage aggregation in atherosclerotic plaques and increasing plaque stability by decreasing cytokine secretion [48]. Furthermore, it has been reported that inhibiting the mTOR signaling pathway can reduce AS in diabetic ApoE^‐/‐^ mice [49]. These findings inspire the hypothesis that appropriately inhibiting the PI3K/AKT/mTOR pathway to restore macrophage autophagy may be a viable target for treating AS patients.

In summary, our current study confirmed that oxLDL/β2GPI/anti‐β2GPI could mediate the inhibition of macrophage autophagy through the TLR4/NK‐κB signaling pathway and PI3K/AKT/mTOR signaling pathway. The inhibitors TAK242, PDTC and LY294002 could partly restore the autophagy inhibited by oxLDL/β2GPI/anti‐β2GPI complex, further suggesting that autophagy could be used as a potential therapeutic target for AS. Despite the valuable insights provided by our study, certain limitations must be acknowledged. First, we did not conduct animal experiments to validate the outcomes derived from our in vitro cell‐based research. Second, our investigation of the signaling pathway was limited to the use of inhibitors, without complementary validation at the genetic level. These deficiencies will be addressed in subsequent studies to enhance the robustness and comprehensiveness of our findings.

## Author Contributions

**Qianqian Wu:** data curation, investigation, project administration, writing–original draft, writing–review and editing. **Guiting Zhang:** data curation, investigation, methodology, writing–original draft, writing–review and editing. **Ting Wang:** conceptualization, funding acquisition, supervision. **Hong Zhou:** conceptualization, funding acquisition, supervision.

## Ethics Statement

This study does not include human participants or animals.

## Conflicts of Interest

The authors declare no conflicts of interest.

## Data Availability

The data that support the findings of this study are available from the corresponding author upon reasonable request.
